# Evaluation of Microleakage Beneath Orthodontic Molar Bands Cemented With Resin‐Modified Glass Ionomer Using Two Enamel Deproteinization Agents

**DOI:** 10.1155/ijod/5723946

**Published:** 2026-02-04

**Authors:** Shabnam Ajami, Maryam Salah, Amirmasoud Rekabpour, Farahnaz Sharafeddin

**Affiliations:** ^1^ Orthodontic Research Center, School of Dentistry, Shiraz University of Medical Sciences, Shiraz, Iran, sums.ac.ir; ^2^ Department of Operative Dentistry, Biomaterials Research Center, School of Dentistry, Shiraz University of Medical Sciences, Shiraz, Iran, sums.ac.ir

**Keywords:** 6% bromelain enzyme, dental microleakage, deproteinization, orthodontic bands

## Abstract

**Objective:**

The aim of this study was to evaluate the microleakage beneath orthodontic bands cemented with resin‐modified glass ionomer (RMGI), following enamel treatment with two different deproteinizing agents in comparison to a control group.

**Materials and Methods:**

Thirty‐six human third molars were used for this cross‐sectional study. The samples were randomly divided into three equal groups of 12. The method of band cementation in each group was as follows: Group I (6% bromelain enzyme as deproteinizer + GC Fuji plus Conditioner + GC Fuji Plus Glass ionomer), Group II (chlorhexidine [CHX] as deproteinizer + GC Fuji Plus Conditioner + Glass ionomer), and Group III (GC Fuji Plus conditioner + GC fuji plus Glass ionomer) as the control group. All samples were sealed and dyed with methylene blue and then exposed to thermocycling. The samples were sectioned and prepared for microleakage evaluation at both occlusal and gingival sides for the enamel–cement interface margins. A stereomicroscope was used to evaluate the amount of dye penetration as the microleakage. Comparison of the microleakage values among the groups was done using the Kruskal–Wallis and Mann–Whitney *U* test as the post hoc test.

**Results:**

The 6% bromelain enzyme deproteinization showed the least amount of microleakage at both occlusal and CEJ sides of the bands (*p*  < 0.05). Group II also showed a better microleakage value at the gingival side in comparison to the control group (*p*  < 0.05).

**Conclusion:**

This study revealed that the pretreatment of enamel with 6% bromelain enzyme significantly reduced the microleakage beneath orthodontic bands cemented with RMGI cement (RMGIC). Improved marginal sealing by 6% bromelain enzyme was significant compared with that of the CHX pretreatment and untreated enamel surfaces.

## 1. Introduction

The term white spot lesion is defined as the first sign of a caries lesion on the enamel that can be detected with the naked eye and presents itself as a milky white opacity, which is located on smooth surfaces [1]. Enamel demineralization and white spot lesions are undesirable, but they are common complications of orthodontic fixed appliance therapy, especially when oral hygiene is poor [2]. Moreover, after orthodontic fixed appliances are attached intraorally, there is a rapid shift in the bacterial flora of the dental plaque. In orthodontic patients, higher bacterial concentrations can lower the pH of the plaque in comparison to non‐orthodontic patients [3].

Less cooperative patients might need supplemental fluoride applications in the form of a varnish or fluoride‐releasing bonding materials. Moreover, fluoride‐releasing composite resins and glass ionomer cements (GICs) have been reported to prevent the decalcification of the enamel, but the bond strength of these materials has been shown to be lower than that of conventional orthodontic resins [4]. Elimination of organic substances from the enamel surface before acid etching increases the resistance against demineralization while providing a better acid etched surface on the enamel [5].

Espinosa et al. [6] suggested that the use of 5.25% NaOCl would increase the bond strength because organic elements are better removed prior to bonding. This is in line with Panchal et al.’s [7] in vitro study, in which 5.25% NaOCl enamel conditioning significantly improved the enamel topography and bracket shear bond strength compared to enamel acid etching alone.

Upon contact with organic material, NaOCl initiates multiple chemical reactions. For example, fatty acids combine with sodium hydroxide to produce soap and glycerol (saponification reaction). Amino acids react with sodium hydroxide to form salt and water (neutralization reaction). Additionally, NaOCl reacts with hypochlorous acid, which creates chloramines and water. These reactions occur concurrently and complementarily, resulting in the liquefaction of organic tissues [8].

Justus et al. [8] were the first to suggest deproteinizing the enamel surface prior to bracket bonding. When 5.25% NaOCl is applied to the enamel surface, it removes the organic elements, enabling the acid etchant to penetrate the enamel more effectively, resulting in significantly increased bracket shear bond strength [8]. Panchal et al. [7] have provided SEM evidence that NaOCl deproteinization could produce a more uniform etching pattern and higher means for shear bond values than phosphoric acid alone. However, NaOCl has an unpleasant taste and the ability to irritate the surrounding tissues, especially in high concentrations [9].

Papain and NaOCl have both been investigated as deproteinizing agents in several studies [7, 10, 11]. Direct comparisons between papain and NaOCl, such as in the study conducted by Panchal et al. [7], suggested promising effects, whereas the role of bromelain has been less frequently explored. Bromelain is a glycoprotein and the major cysteine proteinase among the enzymes present in the stem of the pineapple plant [12]. The enzyme is distinct immunologically and is also found in pineapple [13]. This material has shown notablteeth‐whitening properties and has been utilized as the active ingredient in dentifrices for stain removal, especially when combined with papain. It is available as a dental gel combined with chloramine to remove caries from infected dentin chemomechanically without damaging healthy tissues. In root canal therapy, bromelain has also been used both alone and in combination with chlorhexidine (CHX) as an irrigant. Also, it has demonstrated great promise in orthodontics for deproteinizing the enamel prior to orthodontic bracket bonding [14]. Bromelain is available in various formulations, typically as powders or gels, with concentrations ranging from 3% to 10%. Sharafeddin et al. [15] showed that deproteinization with 6% bromelain enzyme had a higher shear bond strength compared to 10% bromelain enzyme.

Although banding of all teeth has been almost eliminated from fixed orthodontic therapy, banding of the first molars is still necessary most of the time for anchorage in contemporary orthodontics. Therefore, it should be considered that due to the mechanical retention of the orthodontic bands, microleakage beneath them may not be detected for a while, and this would jeopardize the sound enamel under the orthodontic bands, which might lead to white spot lesions [16]. Application of enamel deproteinization may increase the bond of resin‐modified (RMGICs) and decrease the risk of microleakage [17]. This study aimed to evaluate the effect of enamel deproteinization methods on the amount of microleakage beneath the orthodontic bands. The hypothesis was that application of such materials prior to banding could reduce microleakage.

## 2. Materials and Methods

**Figure 1:**
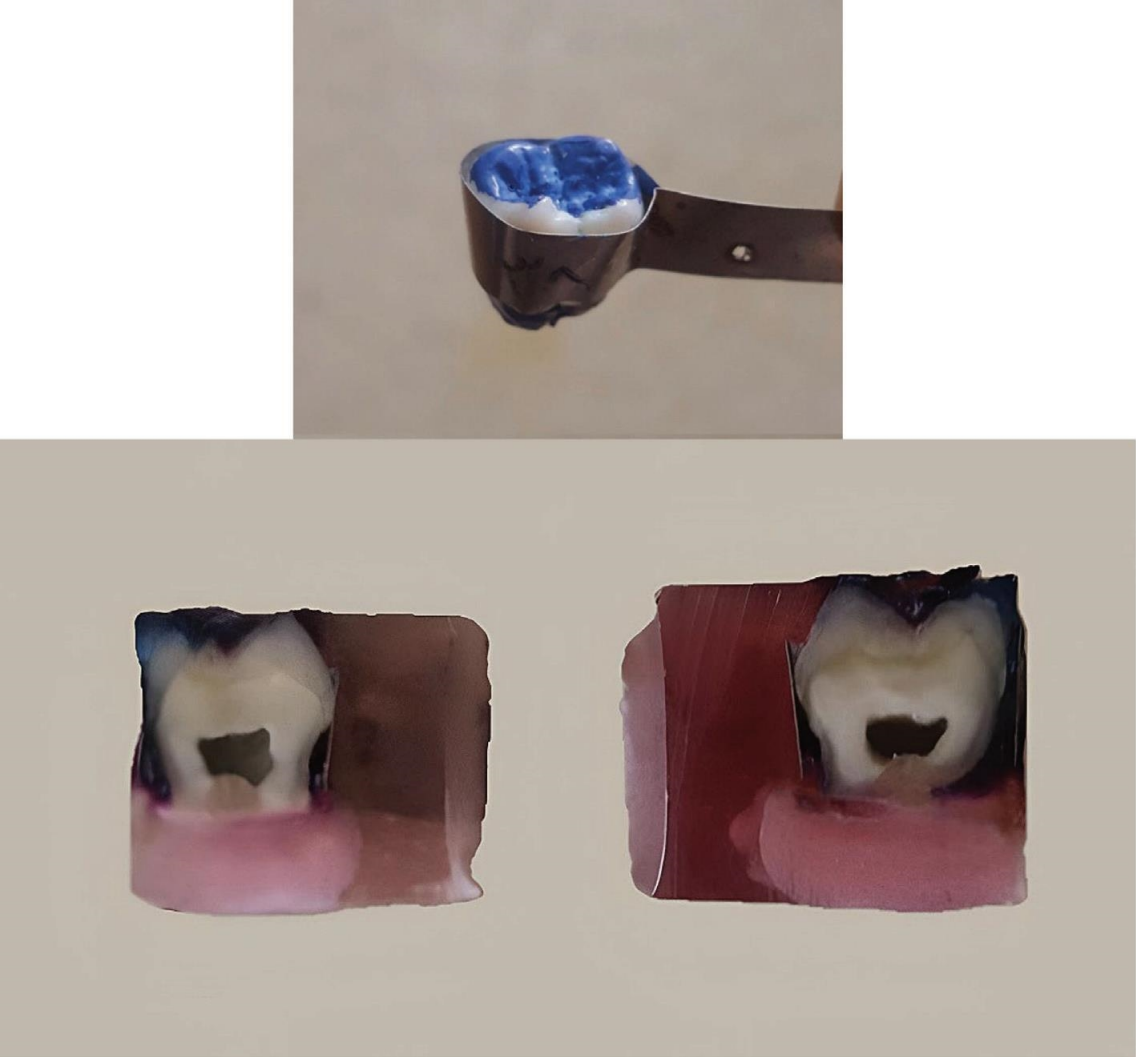
Sample preparation procedure.

A set of 36 non‐carious human third molars, extracted for orthodontic reasons (because of space deficiencies in the posterior segments of the mandibular dental arches in young adults), was collected from patients treated at the Department of Orthodontics, Shiraz University of Medical Sciences, Shiraz, Iran. Ethical approval for this study was obtained from the Ethics Committee of Shiraz University of Medical Sciences (IR.SUMS.REC.1397.653, approved February 13, 2018). All participants provided written informed consent. The samples were stored in distilled water, changed regularly, for up to 1 month after extraction. The surface of the enamel of all the samples was cleaned and polished with pumice paste for 10 s using a rubber prophylactic cap and rinsed with water for 15 s. Before banding, all the teeth were immersed in 1% thymol solution for 1 week for disinfection. First, customized bands were prepared for each sample. Band material (Original Tofflemire Dead Soft Matrix Band, WATER PIK, Mississauga, Canada) was used to fabricate customized bands for each sample and welded with a welding machine (Junior 3000, Dentaurum, Germany). Thereafter, all the root segments of the samples were cut with the diamond disc (NTI Sintered, Double‐sided Diamond Disc, Kahla, Germany) to allow embedding in the polyester resin (Figure 1).

The samples were randomly divided into three equal groups of 12, using random selection. The buccal enamel surfaces of all the samples were lightly polished with pumice to refresh the enamel prior to banding. The three groups received the following enamel surface treatments and adhesive‐application procedures prior to seating and adapting the customized bands around the corresponding teeth.

Group 1: Bromelain enzyme powder (Biozym Scientific GmbH, Olendorf, Germany) was weighed using a balance with an accuracy of ±0.1 mg (GR‐300; A&D Company Ltd., Tokyo, Japan), and 6 g of powder was dissolved in 100 mL of distilled water to obtain a 6% bromelain enzyme solution.

Six percent bromelain enzyme was applied on the refreshed enamel for 1 min, rinsed, and dried for 20 s with oil‐free air spray [15, 17]. GC Fuji PLUS Conditioner (GC Corporation, Tokyo, Japan), a 20% polyacrylic acid solution, was then applied to the enamel surface for 10 s using a microbrush, followed by rinsing with water and drying with oil‐free air for 20 s, based on the manufacturer’s instructions. GC Fuji PLUS RMGIC (GC Corporation, Tokyo, Japan) was applied to the inner surface of the bands.

Group 2: CHX was applied (GLUCO‐CHeX 2% gel, Imen Daroo Novin Co., Tehran, Iran) on the refreshed area for 30 s, rinsed, and dried for 20 s, using oil‐free air spray. Then, GC Fuji PLUS Conditioner was applied as described above, and GC Fuji PLUS RMGIC was used on the inner surface of the bands.

Group 3: The samples were rinsed and dried for 20 s with oil‐free air spray. GC Fuji PLUS Conditioner was applied as previously described, followed by placement of GC Fuji PLUS RMGIC on the inner surface of the customized bands.

The RMGIC (GC Fuji PLUS) used in this study for the banding procedure was self‐curing at room temperature (20°C), with a working time of 2 min and 30 s and a setting time of 4 min and 30 s. Thereafter, thermocycling of the samples was performed between 5 ± 2 and 55 ± 2°C for 500 cycles, with a dwelling time of 30 s and a transfer time of 5 s [18–21]. To prepare the samples for dye penetration, the tooth apices were sealed with sticky wax and rinsed in tap water and air dried. Then, nail varnish was applied to all tooth surfaces, leaving an ~1‐mm window around the occlusal and gingival margins of the bands. The teeth were kept in water as soon as the nail polish dried to minimize dehydration of the samples. All the samples were immersed in 2% methylene blue for 24 h at room temperature. After thorough rinsing with tap water, the superficial dye was removed with a brush and dried. The samples were embedded and fixed into 30 mm (length) x 30 mm (width) x 40 mm (height) polyester resin blocks for 24 h. To determine the dye penetration, a vertical buccolingual cut (through the occlusal and gingival surfaces of each tooth) was made at the middle surface of each banded tooth with a low‐speed diamond disc (Lemgo, Germany).

**Figure 2:**
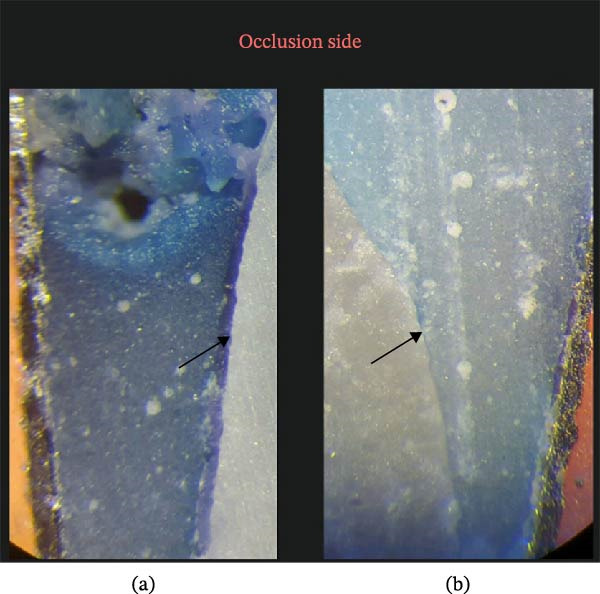
Microleakage at the occlusal margin: (a) presence of microleakage and (b) absence of microleakage.

**Figure 3:**
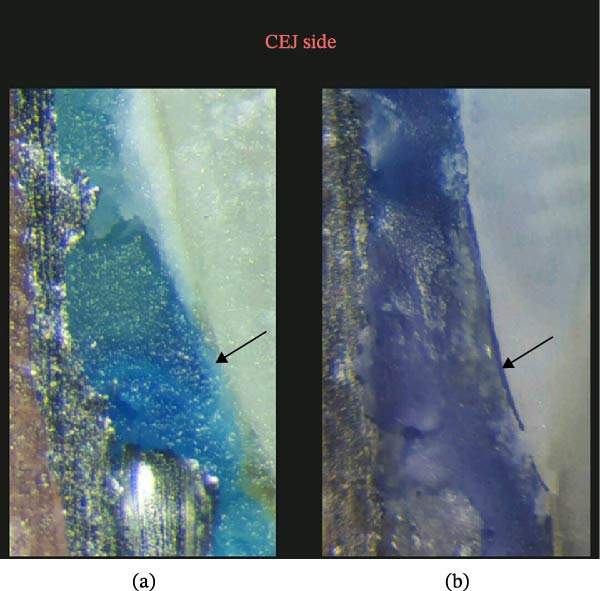
Microleakage at the gingival margin: (a) absence of microleakage and (b) presence of microleakage.

The amount of microleakage was measured in millimeters under a stereomicroscope at ×40 magnification (BS 3060, China‐carton Optical Industries, Pathumthani, Thailand) by direct measurement using the image analyzer software (ScopeImage 9.0, Motic China Group Co., Ltd., Xiamen, China) (Figures 2 and 3).

For each sample, the amount of microleakage was measured in both occlusal and gingival sides along the enamel adhesive interface in millimeters. To evaluate the measurement error, four samples from each group were remeasured and compared with the initial values.

To compare the microleakage values among the groups, Kruskal–Wallis was used and Mann–Whitney *U* test was applied for two‐by‐two comparison between each of the two groups (IBM SPSS statistics, SPSS Version 22). Intra‐examiner error was evaluated using the intraclass correlation coefficient (ICC) statistics. The level of significance was considered at *p*  < 0.05.

## 3. Results

The ICC analysis demonstrated up to 98% reliability, indicating minimal measurement error.

Normality was assessed using Shapiro–Wilk test. The test indicated that the distribution significantly deviated from normal distribution in all groups (all *p*  < 0.05). Therefore, nonparametric Kruskal–Wallis and Mann–Whitney *U* tests were employed to compare the groups.

The level of microleakage differed significantly among the groups (*P* < 0.05). Statistically significant differences were observed in pairwise comparisons of the groups at both the occlusal and gingival margins. At both margins, the control group showed higher microleakage levels compared with the two experimental groups. The lowest microleakage level was observed in the 6% bromelain enzyme‐treated group, which differed significantly from both the control and CHX groups at the occlusal and gingival margins, as given in Tables 1 and 2.

**Table 1:** Pairwise comparison of microleakage values at the occlusal margin among the three groups.

Group	Mean (mm)	Standard deviation	*p* Value
Bromelain acid (*n* = 12)	0.2359	0.15735	0.001 ^∗^
Chlorhexidine (*n* = 12)	0.5862	0.07550	
Bromelain acid (*n* = 12)	0.2359	0.15735	<0.001 ^∗^
Control (*n* = 12)	0.7198	0.10836	
Chlorhexidine (*n* = 12)	0.5862	0.07550	0.48
Control (*n* = 12)	0.7198	0.10836	

^∗^Statistically significant.

**Table 2:** Pairwise comparison of microleakage values at the gingival margin among the three groups.

Group	Mean (mm)	Standard deviation	*p* Value
Bromelain acid (*n* = 12)	0.2881	0.10871	0.007 ^∗^
Chlorhexidine (*n* = 12)	0.4883	0.11466	
Bromelain acid (*n* = 12)	0.2881	0.10871	<0.001 ^∗^
Control (*n* = 12)	0.6853	0.13340	
Chlorhexidine (*n* = 12)	0.4883	0.11466	0.021 ^∗^
Control (*n* = 12)	0.6853	0.13340	

^∗^Statistically significant.

Representative examples of dye penetration at the occlusal margin are shown in Figure 2, demonstrating clear differences in microleakage patterns among the groups.

Pairwise comparisons using the Mann–Whitney *U* test confirmed that the bromelain group had significantly lower microleakage levels compared with both the CHX and control groups at both occlusal and gingival margins ( *p* < 0.001). Additionally, the CHX group exhibited significantly lower microleakage than the control group at the gingival margin (*p* = 0.021); however, this difference was not significant at the occlusal margin (*p* = 0.48).

Figure 3 illustrates dye penetration at the gingival margin, supporting the quantitative findings in Tables 1 and 2.

## 4. Discussion

This study demonstrated that enamel deproteinization with 6% bromelain enzyme prior to band cementation significantly reduced the microleakage compared to 2% CHX and the control group. This finding is clinically relevant, as microleakage beneath orthodontic bands is a known risk factor for enamel demineralization and the development of white spot lesions, particularly in patients with poor oral hygiene [22–25]. While GICs have been widely used for orthodontic bonding due to their fluoride release and chemical adhesion [26], their retention is still limited, especially in the presence of surface debris or plaque [10, 26, 27]. In this study, deproteinization improved the marginal sealing of RMGIC, potentially by enhancing its adaptation to the enamel surfaces. This effect may compensate for the lack of full adaptation between the bands and teeth, which otherwise promotes microleakage.

It seems that chemical debris removal can have its own advantages [25, 28]. Deproteinization of the enamel surface was first introduced by Justus et al. [8]; also, other studies revealed that enamel conditioning with 5.25% NaOCl for 60 s followed by etching could increase the shear bond strength of the brackets bonded with glass ionomer [29]. Justus et al. [8] have also reported that the marginal seal of brackets bonded to the enamel improved.

The result of this study also revealed that enamel deproteinization could enhance the sealing ability of resin‐modified glass ionomers (RMGIs) as the cement for banding the molars.

It is important to highlight that while microleakage evaluation reflects the integrity of the tooth–cement interface and its sealing ability, it does not directly measure the bond strength of the adhesive to the enamel. However, the reduced microleakage may suggest enhanced marginal adaptation, which could indirectly correlate with improved bond durability over time [30].

The findings of this study are consistent with previous studies on chemical deproteinization, showing enhanced interfacial sealing when the enamel was treated with 6% bromelain [15].

Similar improvements in the marginal seal following NaOCl deproteinization were reported by Panchal et al. [7] for self‐cure GIC.

In 2012, 10% papain was introduced as the deproteinizing agent before acid etching, demonstrating better bond strength of the brackets bonded with RMGIC. However, none of these studies evaluated the marginal gap of the resin–enamel interface [10, 31, 32].

In 2015, newer techniques for deproteinization, such as collagenase or bromelain enzyme, were introduced. In adhesive systems, bromelain enzyme could reduce nanoleakage after collagen removal [33]. In addition, the application of bromelain enzyme on the dentin showed better bond strength compared with NaOCl [33].

CHX acts as a matrix metalloproteinase inhibitor and can maintain the resin–dentin bond strength for 6 months by preventing the degradation of the dentin collagen. CHX mouthwash has been shown to have a toxic effect on the fibroblasts, but there is no evidence in the literature for the cytotoxicity of bromelain enzyme [9, 12]. In a study by Sharafeddin and Moraveji [17], 6% bromelain was applied for 60 s, demonstrating favorable effects on the bond strength, but longer application durations have not been investigated. In the present study, a greater seal was observed when enamel deproteinization was achieved, using 6% bromelain enzyme compared to CHX gel. The comparison of these two materials with NaOCl showed that they both had better taste and odor and less tissue reaction. The bond between orthodontic attachments and the enamel surface might be compromised by the acquired pellicle at the time of bonding [34].

It covers the enamel surface before the first layer of oral biofilm attaches and plays a minimal homeostasis role on the tooth enamel. It is formed as the selective adsorption of proteins and peptides in the oral fluid [35].

The presence of this layer can be assumed when comparing the control group with deproteinized groups as the amount of microleakage is significantly higher in the control group. RMGIC and acid‐modified composites showed acceptable properties. They are both capable of releasing fluoride and inhibiting microleakage growth [36].

Although RMGICs offer improved handling and faster setting compared to conventional GICs, they are subject to polymerization shrinkage due to their resin content. This shrinkage may compromise the marginal seal and contribute to microleakage, especially under thermal stress. The presence of 2‐hydroxyethyl methacrylate (HEMA) in RMGICs is associated with increased water absorption and dimensional changes, which may further influence the sealing ability at the tooth–cement interface [37, 38].

Conventional acid‐based GICs have demonstrated superior marginal integrity in some studies, often showing negligible microleakage [39]. Including a control group with conventional GIC, with and without thermo‐curing, could, therefore, have provided more comprehensive comparisons of marginal seal performance.

From a clinical perspective, the adhesive strength required for reliable cementation of orthodontic bands and brackets typically ranges between 6 and 8 MPa [22]. While conventional GICs generally exhibit lower bond strength, they are valued for their excellent fluoride release and favorable biocompatibility [26]. In contrast, RMGICs provide improved mechanical properties and clinically acceptable bond strength, making them suitable for orthodontic banding applications; however, their resin component predisposes them to polymerization shrinkage [27]. These performance trade‐offs should be carefully considered in clinical decision‐making.

In the present study, the lowest microleakage levels were observed in the bromelain group, supporting the beneficial role of deproteinization in enhancing the marginal adaptation of RMGIC to enamel surfaces.

Also, this result supports the findings of Pithon et al. [14], who demonstrated that application of bromelain increased the bracket shear bond strength, and Chauhan et al. [33], who indicated that it was a better choice of deproteinizing agent without drawbacks. The adhesion process with NaOCl demonstrated a lower bond strength. The decrease in the mechanical properties of adhesives may be caused by the presence of oxygen after disintegrating NaOCl into NaCl and O2. Release of oxygen during polymerization might inhibit the process [40].

The results of the present study bring new perspectives while considering band cementation during fixed orthodontic treatments. Deproteinization led to a decrease in the microleakage, which would increase the retention of bands, especially under heavy forces such as those applied with headgear. Although we know that proper band selection is the key to successful banding in orthodontics, enamel pretreatments can also be helpful. However, this study had some limitations, such as the small sample size and lack of long‐term evaluation since this was an in vitro and cross‐sectional study. As a result, further clinical evaluations are necessary to enable a comprehensive understanding of the mechanisms by which deproteinizing agents act.

Another limitation of the present study is the absence of a conventional GIC control group. Including classic GIC—with and without thermo‐curing—could have provided a more comprehensive comparison, as conventional GICs have been shown to exhibit superior marginal sealing and lower microleakage compared to RMGICs [39]. Future investigations should include these materials to further validate the performance of deproteinizing agents with various cement types.

## 5. Conclusion

This study revealed that the pretreatment of enamel with 6% bromelain enzyme significantly reduced the microleakage beneath orthodontic bands cemented with RMGIC. Improved marginal sealing by 6% bromelain enzyme was significant compared with that of the CHX pretreatment and untreated enamel surfaces.

## Funding

No funding was received for this manuscript.

## Ethics Statement

Ethical approval for this study was provided by the ethics committee of Shiraz University of Medical Sciences, Shiraz, on February 13, 2018, with the code of IR.SUMS.REC.1397.653.

## Conflicts of Interest

The authors declare no conflicts of interest.

## Data Availability

The data that support the findings of this study are available upon request from the corresponding author.

## References

[1] Sampson V. and Sampson A. , Diagnosis and Treatment Options for Anterior White Spot Lesions, British Dental Journal. (2020) 229, no. 6, 348–352, 10.1038/s41415-020-2057-x. 32978577

[2] Linjawi A. I. , Sealants and White Spot Lesions in Orthodontics: A Review, The Journal of Contemporary Dental Practice. (2020) 21, no. 7, 808–814, 10.5005/jp-journals-10024-2882. 33020368

[3] Freitas A. O. A. , Marquezan M. , Nojima M. C. G. , Alviano D. S. , and Maia L. C. , The Influence of Orthodontic Fixed Appliances on the Oral Microbiota: A Systematic Review, Dental Press Journal of Orthodontics. (2014) 19, no. 2, 46–55, 10.1590/2176-9451.19.2.046-055.oar, 2-s2.0-84904399472. PMC4296609 24945514

[4] Khan A. R. , Fida M. , and Gul M. , Decalcification and Bond Failure Rate in Resin Modified Glass Ionomer Cement versus Conventional Composite for Orthodontic Bonding: A Systematic Review & Meta-Analysis, International Orthodontics. (2020) 18, no. 1, 32–40, 10.1016/j.ortho.2019.10.003. 31882396

[5] Abreu L. G. , Paiva S. M. , Pretti H. , Lages E. M. B. , Júnior J. B. N. , and Ferreira R. A. N. , Comparative Study of the Effect of Acid Etching on Enamel Surface Roughness Between Pumiced and Non-Pumiced Teeth, Journal of International Oral Health: JIOH. (2015) 7, no. 9, 1–6. PMC4589700 26435607

[6] Espinosa R. , Valencia R. , Uribe M. , Ceja I. , and Saadia M. , Enamel Deproteinization and Its Effect on Acid Etching: An In Vitro Study, Journal of Clinical Pediatric Dentistry. (2008) 33, no. 1, 13–19, 10.17796/jcpd.33.1.ng5462w5746j766p, 2-s2.0-60849086210. 19093646

[7] Panchal S. , Ansari A. , Jain A. K. , and Garg Y. , Effects of Different Deproteinizing Agents on Topographic Features of Enamel and Shear Bond Strength: An In Vitro Study, Journal of Orthodontic Science. (2019) 8, no. 1, 10.4103/jos.JOS_26_19, 17. 31649897 PMC6803782

[8] Justus R. , Cubero T. , Ondarza R. , and Morales F. , A New Technique With Sodium Hypochlorite to Increase Bracket Shear Bond Strength of Fluoride-Releasing Resin-Modified Glass Ionomer Cements: Comparing Shear Bond Strength of Two Adhesive Systems With Enamel Surface Deproteinization Before Etching, Seminars in Orthodontics, 2010, Elsevier, Philadelphia, 55–63.

[9] Bin-Shuwaish M. S. , Effects and Effectiveness of Cavity Disinfectants in Operative Dentistry: A Literature Review, Journal of Contemporary Dental Practice. (2016) 17, no. 10, 867–879, 10.5005/jp-journals-10024-1946, 2-s2.0-85012890279. 27794161

[10] Pithon M. M. , de Souza Ferraz C. , and do Couto de Oliveira G. , et al.Effect of 10% Papain Gel on Enamel Deproteinization before Bonding Procedure, The Angle Orthodontist. (2012) 82, no. 3, 541–545, 10.2319/062911-423.1, 2-s2.0-84861325387. 22077189 PMC8865809

[11] Khatib M. S. , Devarasanahalli S. V. , Aswathanarayana R. M. , Venkateswara A. H. , and Nadig R. R. , Microtensile Bond Strength of Composite Resin Following the use of Bromelain and Papain as Deproteinizing Agents on Etched Dentin: An In Vitro Study, International Journal of Clinical Pediatric Dentistry. (2020) 13, no. 1, 43–47, 10.5005/jp-journals-10005-1743. 32581478 PMC7299883

[12] Azarkan M. , Maquoi E. , and Delbrassine F. , et al.Structures of the Free and Inhibitors-Bound Forms of Bromelain and Ananain From *Ananas comosus* Stem and In Vitro Study of Their Cytotoxicity, Scientific Reports. (2020) 10, no. 1, 10.1038/s41598-020-76172-5, 19570. 33177555 PMC7658999

[13] Ramli A. N. M. , Manas N. H. A. , Hamid A. A. A. , Hamid H. A. , and Illias R. M. , Comparative Structural Analysis of Fruit and Stem Bromelain from *Ananas comosus* , Food Chemistry. (2018) 266, 183–191, 10.1016/j.foodchem.2018.05.125, 2-s2.0-85047972721. 30381175

[14] Pithon M. M. , Campos M. S. , and da Silva Coqueiro R. , Effect of Bromelain and Papain Gel on Enamel Deproteinisation Before Orthodontic Bracket Bonding, Australasian Orthodontic Journal. (2016) 32, no. 1, 23–30, 10.21307/aoj-2020-109. 27468588

[15] Sharafeddin F. , Jowkar Z. , and Safari M. , Effects of Different Concentrations of Bromelain and Papain Enzymes on Shear Bond Strength of Composite Resin to Deep Dentin Using an Etch-and-Rinse Adhesive System, Dental and Medical Problems. (2024) 61, no. 1, 85–91, 10.17219/dmp/133404. 38441350

[16] Goje S. K. , Sangolgi V. K. , Neela P. , and Lalita C. H. , A Comparison of Resistance to Enamel Demineralization After Banding With Four Orthodontic Cements: An In Vitro Study, Journal of Indian Orthodontic Society. (2012) 46, no. 3, 141–147, 10.1177/0974909820120305.

[17] Sharafeddin F. and Moraveji P. , Comparison the Effect of Bromelain Enzyme, Phosphoric Acid, and Polyacrylic Acid Treatment on Microleakage of Composite and Glass Ionomer Restorations, Journal of Dentistry (Shiraz, Iran). (2022) 23, no. 1 Suppl, 175–182, 10.30476/DENTJODS.2021.88737.1355. 36380843 PMC9652053

[18] Arhun N. , Arman A. , Çehreli S. B. , Arıkan S. , Karabulut E. , and Gülşahı K. , Microleakage Beneath Ceramic and Metal Brackets Bonded With a Conventional and an Antibacterial Adhesive System, The Angle Orthodontist. (2006) 76, no. 6, 1028–1034, 10.2319/101805-368, 2-s2.0-33750600011. 17090167

[19] Davari A. , Yassaei S. , and Zoghi H. , Effect of Thermocycling on Shear Bond Strength of a Conventional Etch and Rinse Adhesive System, Iranian Journal of Orthodontics. (2011) 6, no. 3, 17–21.

[20] Pakshir H. and Ajami S. , Effect of Enamel Preparation and Light Curing Methods on Microleakage Under Orthodontic Brackets, Journal of Dentistry (Tehran, Iran). (2015) 12, no. 6, 436–446. 26884778 PMC4754570

[21] Sokucu O. , Siso S. H. , Ozturk F. , and Nalcaci R. , Shear Bond Strength of Orthodontic Brackets Cured With Different Light Sources Under Thermocycling, European Journal of Dentistry. (2010) 4, no. 3, 257–262, 10.1055/s-0039-1697837. 20613913 PMC2897858

[22] Bishara S. E. and Ostby A. W. , White Spot Lesions: Formation, Prevention, and Treatment, Seminars in Orthodontics, 2008, Elsevier, Philadelphia, 174–182.

[23] Lazar L. , Vlasa A. , and Beresescu L. , et al.White Spot Lesions (WSLs)—Post-Orthodontic Occurrence, Management and Treatment Alternatives: A Narrative Review, Journal of Clinical Medicine. (2023) 12, no. 5, 10.3390/jcm12051908, 1908. 36902696 PMC10003622

[24] Behnan S. M. , Arruda A. O. , González-Cabezas C. , Sohn W. , and Peters M. C. , In-Vitro Evaluation of Various Treatments to Prevent Demineralization Next to Orthodontic Brackets, American Journal of Orthodontics and Dentofacial Orthopedics. (2010) 138, no. 6, 712.e1–712.e7, 10.1016/j.ajodo.2010.05.014, 2-s2.0-85058720593. 21130326

[25] Tasios T. , Papageorgiou S. N. , Papadopoulos M. A. , Tsapas A. , and Haidich A. B. , Prevention of Orthodontic Enamel Demineralization: A Systematic Review With Meta-Analyses, Orthodontics & Craniofacial Research. (2019) 22, no. 4, 225–235, 10.1111/ocr.12322, 2-s2.0-85073083127. 31081584

[26] Fricker J. P. , Therapeutic Properties of Glass-Ionomer Cements: Their Application to Orthodontic Treatment, Australian Dental Journal. (2022) 67, no. 1, 12–20, 10.1111/adj.12888. 34762310

[27] Pithon M. M. , Dos Santos R. L. , de Oliveira M. V. , Ruellas A. C. O. , and Romano F. L. , Metallic Brackets Bonded With Resin-Reinforced Glass Ionomer Cements Under Different Enamel Conditions, The Angle Orthodontist. (2006) 76, no. 4, 700–704. 16808580 10.1043/0003-3219(2006)076[0700:MBBWRG]2.0.CO;2

[28] Mesquita J. A. , Lacerda-Santos R. , Sampaio G. A. , Godoy G. P. , Nonaka C. F. , and Alves P. M. , Evaluation In Vivo of Biocompatibility of Different Resin-Modified Cements for Bonding Orthodontic Bands, Anais da Academia Brasileira de Ciências. (2017) 89, no. 3 suppl, 2433–2443, 10.1590/0001-3765201720170329, 2-s2.0-85034967509. 29091110

[29] Pereira T. B. J. , Jansen W. C. , Pithon M. M. , Souki B. Q. , Tanaka O. M. , and Oliveira D. D. , Effects of Enamel Deproteinization on Bracket Bonding With Conventional and Resin-Modified Glass Ionomer Cements, The European Journal of Orthodontics. (2013) 35, no. 4, 442–446, 10.1093/ejo/cjs006, 2-s2.0-84880971151. 22379131

[30] Van Meerbeek B. , De Munck J. , and Yoshida Y. , et al.Adhesion to Enamel and Dentin: Current Status and Future Challenges, Operative Dentistry. (2003) 28, no. 3, 215–235. 12760693

[31] Pithon M. M. , Ferraz C. S. , Oliveira G. D. C. , and Santos A. M. D. , Effect of Different Concentrations of Papain Gel on Orthodontic Bracket Bonding, Progress in Orthodontics. (2013) 14, no. 1, 1–5, 10.1186/2196-1042-14-22, 2-s2.0-84884581656. 24325920 PMC4384916

[32] Amaral F. B. , Florio F. M. , Ambrosano G. B. , and Basting R. T. , Morphology and Microtensile Bond Strength of Adhesive Systems to In Situ-Formed Caries-Affected Dentin After the use of a Papain-Based Chemomechanical Gel Method, American Journal of Dentistry. (2011) 24, 13–19. 21469401

[33] Chauhan K. , Basavanna R. S. , and Shivanna V. , Effect of Bromelain Enzyme for Dentin Deproteinization on Bond Strength of Adhesive System, Journal of Conservative Dentistry. (2015) 18, no. 5, 360–363, 10.4103/0972-0707.164029, 2-s2.0-84941108334. 26430297 PMC4578178

[34] Retamoso L. B. , Collares F. M. , Ferreira E. S. , and Samuel S. M. W. , Shear Bond Strength of Metallic Brackets: Influence of Saliva Contamination, Journal of Applied Oral Science. (2009) 17, no. 3, 190–194, 10.1590/S1678-77572009000300011, 2-s2.0-67649992762. 19466249 PMC4399530

[35] Siqueira W. L. , Bakkal M. , Xiao Y. , Sutton J. N. , and Mendes F. M. , Quantitative Proteomic Analysis of the Effect of Fluoride on the Acquired Enamel Pellicle, PLoS ONE. (2012) 7, no. 8, 10.1371/journal.pone.0042204, 2-s2.0-84864759827. PMC3411614 22870302

[36] Neti B. , Sayana G. , Muddala L. , Mantena S. R. , Yarram A. , and Harsha G. , Fluoride Releasing Restorative Materials: A Review, International Journal of Dental Materials. (2020) 02, no. 1, 19–23, 10.37983/IJDM.2020.2104.

[37] Sidhu S. K. and Nicholson J. W. , A Review of Glass-Ionomer Cements for Clinical Dentistry, Journal of Functional Biomaterials. (2016) 7, no. 3, 10.3390/jfb7030016, 16. 27367737 PMC5040989

[38] Czarnecka B. and Nicholson J. W. , Ion Release by Resin-Modified Glass-Ionomer Cements Into Water and Lactic Acid Solutions, Journal of Dentistry. (2006) 34, no. 8, 539–543, 10.1016/j.jdent.2005.08.007, 2-s2.0-33747058969. 16504366

[39] Abd El Halim S. and Zaki D. , Comparative Evaluation of Microleakage Among Three Different Glass Ionomer Types, Operative Dentistry. (2011) 36, no. 1, 36–42, 10.2341/10-123-LR, 2-s2.0-79955642443. 21488727

[40] Lai S. , Mak Y. , and Cheung G. , et al.Reversal of Compromised Bonding to Oxidized Etched Dentin, Journal of Dental Research. (2001) 80, no. 10, 1919–1924, 10.1177/00220345010800101101, 2-s2.0-0034767685. 11706952

